# Nutritional content, protein quantity, protein quality and carbon footprint of plant-based drinks and semi-skimmed milk in the Netherlands and Europe

**DOI:** 10.1017/S1368980022000453

**Published:** 2022-05

**Authors:** Cécile M Singh-Povel, Martine P van Gool, Ana Paulina Gual Rojas, Marjolijn CE Bragt, Anne J Kleinnijenhuis, Kasper A Hettinga

**Affiliations:** 1 FrieslandCampina, Stationsplein 4, Amersfoort LE 3818, The Netherlands; 2 Blonk Consultants, Gouda, The Netherland; 3 Triskelion, Utrecht, The Netherlands; 4 Wageningen University and Research, Wageningen, The Netherlands

**Keywords:** Bovine milk, Plant-based drinks, Nutritional value, Protein, Essential amino acids, Carbon footprint, Sustainable diet

## Abstract

**Objective:**

To compare the nutritional composition of bovine milk and several plant-based drinks with a focus on protein and essential amino acid content and to determine the ratio of essential amino acids to greenhouse gas emission.

**Design:**

Nutritional information on the label was extracted for semi-skimmed milk, soy, oat, almond, coconut and rice drink from the Innova database between January 2017 and March 2020 for the Netherlands, Belgium, Germany, Spain, Italy and Sweden. Protein and amino acids were measured and carbon footprint was calculated for a selection of Dutch products. Protein quality was determined by calculating the contribution to the WHO essential amino acids requirements.

**Setting:**

The bovine milk and plant-based drinks market in Netherlands, Belgium, Germany, Spain, Italy and Sweden.

**Participants:**

Semi-skimmed bovine milk and soy, oat, almond, coconut and rice drink.

**Results:**

Nutritional label information was collected for 399 products. Milk naturally contains many micronutrients, e.g. vitamin B_2_, B_12_ and Ca. Approximately 50 % of the regular plant-based drinks was fortified with Ca, whereas the organic plant-based drinks were mostly unfortified. Protein quantity and quality were highest in milk. Soy drink had the best protein quality to carbon footprint ratio and milk came second.

**Conclusions:**

The nutrition – climate change balance presented in this study, is in line with previous literature, which shows that semi-skimmed bovine milk and fortified soy drink deserve a place in a sustainable diet.

## Graphical abstract

**Figure:**
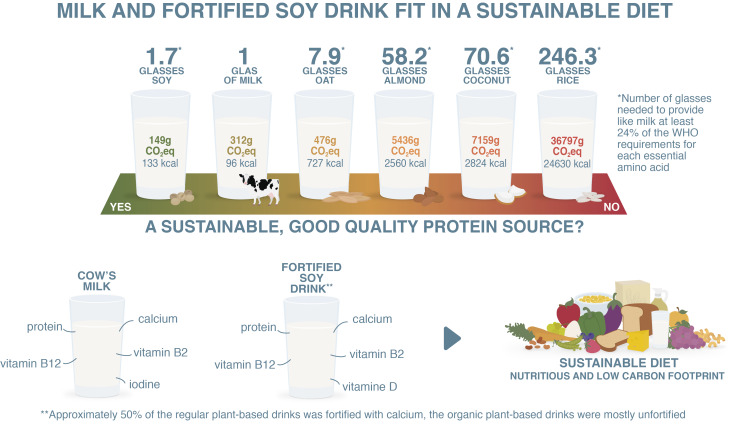

To combat global warming, it is important to reduce greenhouse gas emissions. The food system as a whole is responsible for about 25 % of greenhouse gas emissions worldwide^(1)^. The greenhouse gas emission of the food system can be decreased by a combination, of, among others: (1) improved production practices; (2) reduced waste and (3) dietary changes^(2)^.

One of the most frequently proposed dietary change measures is to decrease consumption of animal-based foods, particularly meat, combined with the advice to increase the consumption of plant-based foods, such as vegetables, fruits, legumes and nuts. The call to increase consumption of plant-based foods has also lifted the market of plant-based drinks, such as soy, oat, almond, coconut and rice drink^(3)^. Although the average Dutch daily consumption levels of plant-based drinks are relatively low (7·8 g/d)^(4)^, consumption of these drinks is expected to increase.

Given the increasing consumption of plant-based drinks in children and adults, it is important to determine the nutritional impact of replacing bovine milk by these drinks. Milk naturally contains many micronutrients, such as Ca, vitamin B_2_, vitamin B_12_ and I. In the Western world, bovine milk provides over 50 % of total Ca intake and around 30 % of the total intake of vitamin B_2_, vitamin B_12_ and I^(5)^. Therefore, bovine milk makes a substantial contribution to micronutrient intake. Plant-based drinks, do naturally not, or only in very low levels, contain the above-mentioned micronutrients^(6)^. Levels of those micronutrients in plant-based drinks are thus mostly dependent on fortification practices^(7)^. Hence, it is important to investigate how prevalent those fortification practices are. Furthermore, it is important to consider potential differences in nutrient bioavailability between these types of drinks and *v*. bovine milk^(8,9)^.

In addition to differences in micronutrient composition, available literature shows that most plant-based drinks, except for soy drink, are significantly lower in protein content than bovine milk^(3,7,10–12)^. On top of that, it has been shown that the protein quality of plant-based ingredients, such as soy and rice protein isolates, is lower compared with dairy ingredients, such as milk protein concentrate, mainly due to a less favourable essential amino acids profile^(11)^. However, information on the actual amount of essential amino acids present in plant-based consumer drinks is lacking. As milk provides approximately 10 % of total protein intake in the Netherlands^(4,5)^ and as plant-based drinks are positioned as dairy alternatives, it is important to determine the contribution of these drinks to the required intake levels for all essential amino acids separately^(13)^. This information on protein quantity and quality is especially relevant for dietary advises to population groups with increased protein need and/or low protein intake such as elderly^(14)^, children^(15)^ and people who eat no or limited animal foods^(16)^. Furthermore, essential amino acids should be considered in the nutritional assessment to determine the position of bovine milk and plant-based drinks in a sustainable diet, i.e. a healthy diet, with a low environmental impact, which also accounts for economic and social factors^(17)^. As proposed sustainable diets are generally accepted to be low in high-quality meat protein^(2)^, protein (quality) is of concern in such sustainable diets advise. To be able to judge which drinks are a sustainable source of protein, one should determine the balance between the protein and essential amino acids provided, environmental impact and affordability. Insights in this balance can be generated by calculating the ratio of essential amino acids to parameters like greenhouse gas emission and price.

The first objective of this article is to compare the nutritional composition of bovine milk and several plant-based drinks with a focus on protein and essential amino acid content. Furthermore, insight in the prevalence of fortification practices for the different plant-based drinks will be generated. The second objective is to determine the ratio of essential amino acids to greenhouse gas emission and price for bovine milk and for plant-based drinks. This information will help to determine the place of bovine milk and plant-based drinks in current and future healthy, sustainable diets.

## Materials and methods

### Products

Nutritional information derived from food labels was collected for the most prevalent bovine milk type, semi-skimmed milk, as well as for the most prevalent plant-based drinks, being soy, oat, almond, coconut and rice drink. For the plant-based drinks regular, organic and unsweetened varieties were included. Nutritional information on the label was obtained for a cross-section of European countries: the Netherlands, Belgium, Germany, Spain, Italy and Sweden.

Protein- and individual amino acids content were measured in a selection of commercially available products in the Netherlands. The selected products were widely available, in different Dutch supermarkets, had data available to make carbon footprint calculations and had a macronutrient composition representative for their category. For semi-skimmed bovine milk, as well as for the regular varieties of soy, oat, almond and coconut drink, a ultra-heat treatment (UHT) and pasteurised product was included. For rice drink only, a UHT product was available.

Carbon footprint was calculated for 1 UHT drink per regular plant-based type and for 1 UHT semi-skimmed bovine milk.

### Nutritional information on the label: database search

Nutritional information on the labels was obtained from the Innova Database^(18)^. The Innova Database contains labelling information of consumer food products from over 75 countries. The database is continuously updated with new products on the market and gives a representative overview of all different brands of food products (both A-brands and private label) available. A search was done for products in the categories ‘dairy alternative drinks’ and ‘unflavoured milk’, which were entered into the database between January 2017 and March 2020. Next, the different brands of semi-skimmed bovine milk, as well as the conventional organic and unsweetened varieties of the plant-based drinks, were manually selected. From all selected products, information was retrieved on energy (kJ), total fat saturated fat, protein, total carbohydrate, sugar, vitamin B_2_, vitamin B_12_, vitamin D, Ca and I content.

For each product type in a country, average values were calculated for energy content and macronutrient content (g/100 g). If 3 or more brands per product type were available, the sd was also calculated. For the plant-based drinks, the percentage of products fortified with vitamin B_2_, vitamin B_12_, vitamin D, Ca and/or I was calculated.

To calculate sugar content, a distinction was made between free sugars and non-free sugars as stated in the WHO guideline, ‘sugar intake for adults and children’^(19)^.

### Protein quantity and quality analyses

All chemicals used were of analytical grade, unless stated otherwise.

#### Protein content determination

The protein content of the products was determined by Kjeldahl^(20,21)^. The protein content is calculated using the nitrogen conversion factor, depending on the protein’s origin (See Table 1). For each product type, protein content was determined for 3 different products, each from a different production batch; each analysis was performed in duplicate. An average protein content and sd was calculated for each product.

**Table 1:** Nitrogen to protein conversion factors for several food types, as suggested by Jones in 1941

Protein	Factor
Milk^(48)^	6·38
Oats^(48)^	5·83
Almonds^(49)^	5·18
Rice^(48)^	5·95
Soybean^(48)^	5·71
Coconut^(50)^	5·31

#### Essential amino acid composition determination

Due to the low protein concentration, samples of coconut and rice drink were freeze-dried prior to analyses. The amino acids cystine/cysteine, histidine, isoleucine, leucine, lysine, methionine, phenylalanine, threonine, tyrosine and valine were quantified using ion chromatography, post-column derivatisation with ninhydrin and detection using UV-VIS absorption, after (oxidation and) hydrolysis. The applied amino acid analyzer was a Hitachi L-8900 (Hitachi, Tokyo, Japan). After oxidation, both cysteine and cystine are converted to cysteic acid. The results are expressed as cystine. The amino acid tryptophan was quantified using ultra-performance liquid chromatography and fluorescence detection (Waters Acquity), after hydrolysis. If the concentration of an essential amino acid was below the detection limit, the detection limit was used in further calculations, thereby potentially overestimating the result.

The amino acid composition per gram of protein is not expected to differ significantly per product batch. Therefore, amino acid composition was determined in duplo for one product per product type. Amino acid content was standardised to protein content reported on the label, except for soy and oat drink, for which protein content on the label was reported without any decimals, and therefore, measured protein values were used. The sd for the amino acid composition was calculated based on both variation in protein content and known variation from the amino acid composition determination.

For each product type, the contribution of each essential amino acid in 1 glass (200 ml) of product to the WHO requirements for essential amino acids intake^(8)^ was calculated for an adult with an average weight of 70 kg. Afterwards the amino acid with the lowest contribution was determined, as this amino acid would be rate limiting for protein synthesis.

### Calculation of price per product based on essential amino acid content

Price information of the products was obtained from the market leader supermarket in the Netherlands, Albert Heijn (9th June 2020).

### Calculation of carbon footprint per product based on essential amino acid content

A Life Cycle Assessment (LCA) with system boundaries from cradle to grave was conducted to calculate carbon footprint. LCA is a framework that allows identifying the environmental impact of a product across its whole life cycle. This means that all stages in the products’ life, i.e., raw milk or raw ingredients, processing in the factory, packaging, distribution, retail and use, were taken into account to calculate the related carbon footprint. The LCA system boundaries were set in alignment to the Product Environmental Footprint Category Rules for Dairy products (PEFCR Dairy)^(22)^ and general Product Environmental Footprint (PEF) guidance^(23)^. The impact assessment method used was IPCC 2013^(24)^. Background databases used were Agri-Footprint 4·0 and Ecoinvent 3·5. For all calculations, it was assumed that all products were sold and consumed in The Netherlands. For semi-skimmed milk, primary activity data were used to calculate the carbon footprint of raw milk production, processing and packaging. For the 5 commercially available regular plant-based drinks, information on product compositions, as well as publicly available data on the production process, such as energy consumption, electricity mix and origin of ingredients^(25–29)^, were used to calculate the carbon footprint related to the formulation, processing and packaging. Ingredients such as stabilisers, emulsifiers and vitamins constituted <1 % of the plant-based drink composition by mass. Their contribution to the carbon footprint was considered negligible and in alignment with PEF guidance, a cut-off was applied, and these ingredients were not considered for the LCA. For both semi-skimmed milk and plant-based drinks EU default values were used^(22)^ to calculate the carbon footprint related to the life cycle stages of transport, distribution, retail and use. Food losses in distribution and retail are modelled based on default values provided by PEFCR Dairy (5 %) and are assumed the same for plant-based drinks. At the use stage, for both semi-skimmed milk and plant-based drinks, it was assumed that they were stored at ambient temperature and were refrigerated for 5 d after opening. Furthermore, a food loss of 5 % at the end consumer was assumed.

### Statistical analyses

The statistical analysis was performed through the IBM SPSS statistics (version 24). Significant difference between means was calculated by ANOVA and afterwards pairwise comparisons were made with the Tukey post-hoc test. *P* < 0·05 was considered significant.

## Results

### Nutrition label information

Information on the nutrition label for bovine milk, as well as soy, oat, almond, coconut and rice drink was collected for 399 products in total. For regular, bovine semi-skimmed milk, data of 7–30 unique brands per country was collected. For organic bovine milk, 3–8 unique brands per country were included. For the regular types of plant-based drinks, information of 3–11 unique brands per country, per type was collected. For the organic plant-based drinks this ranged from 0 to 13 brands per country, per type. The unsweetened plant-based drinks included 0–10 brands per country, per type (Table 2).

**Table 2:** Macronutrient composition of 100 g regular plant-based drinks or semi-skimmed bovine milk, as displayed on the nutritional label

	Brands (*n*)	Energy (kJ)		Fat (g)		Saturated fat (g)		Protein (g)		Carbohydrate (g)		Free sugar (g)		Lactose (g)	
		Mean	sd	Mean	sd	Mean	sd	Mean	sd	Mean	sd	Mean	sd	Mean	sd
Soy drink															
Netherlands	4	176	29	2·2	1	0·3	0·1	3·0	0	2·3	0·4	2·3	0·4	0	
Belgium	5	180	25	2·1	0·9	0·4	0·1	3·1	0·2	2·6	0·8	2·6	0·7	0	
Germany	4	167	8	2	0·2	0·4	0·2	3·4	0·2	2	0·7	1·3	0·9	0	
Italy	11	172	25	1·9	0·4	0·4	0·1	3·2	0·3	2·6	1	2·2	1·2	0	
Spain	6	172	21	1·7	0·1	0·3	0	3·1	0·0	3·4	0·6	2·6	1	0	
Sweden	6	180	17	2·2	0·4	0·4	0·1	3·4	0·3	2·2	0·9	1·7	1·1	0	
Average		176	21	2·0	0·5	0·4	0·1	3·2	0·2	2·5	0·9	2·1	1·0	0	
Oat drink															
Netherlands	6	213	42	1·9	1	0·3	0·1	0·9	0·3	8·2	1·4	5·2	0·3	0	
Belgium	7	192	25	1·6	0·3	0·2	0·1	0·8	0·5	7	1·3	3·4	1·5	0	
Germany	7	201	38	1·1	0·3	0·2	0·1	0·7	0·6	8·5	1·5	4·8	0·7	0	
Italy	8	192	29	1·2	0·2	0·2	0·1	0·7	0·5	7·7	1·5	5	2·4	0	
Spain	7	188	38	1·1	0·4	0·2	0·1	0·9	0·7	7·5	1·8	4·9	2·2	0	
Sweden	9	184	25	1·2	0·4	0·1	0·1	0·9	0·5	6·9	1	3·2	1·4	0	
Average		195	38	1·3	0·5	0·2	0·1	0·8	0·5	7·5	1·9	4·4	1·6	0	
Almond drink															
Netherlands	6	167	84	1·6	0·9	0·2	0·1	0·5	0·2	6·2	6·3	3·4	1·7	0	
Belgium	7	109	46	1·6	0·9	0·2	0·1	0·4	0·2	2·1	1·2	2·1	1·3	0	
Germany	7	142	59	1·9	0·9	0·4	0·5	1	0·5	2·9	2·5	2·6	2·6	0	
Italy	9	222	138	2·3	1·3	0·3	0·1	0·7	0·5	6·1	5·8	1·8	1·1	0	
Spain	10	138	63	1·8	0·9	0·3	0·2	0·6	0·4	3·3	2·2	2·8	2·1	0	
Sweden	3	151	126	2·4	1·9	0·3	0·2	0·8	0·6	2·7	2·5	2·2	2	0	
Average		155	88	2·2	2·3	0·3	0·2	0·6	0·4	3·4	3·4	2·7	2·2	0	
Coconut drink															
Netherlands	5	96	13	1·1	0·5	1·1	0·5	0·1	0·1	2·9	0·8	1·9	0·2	0	
Belgium	3	138	71	1·7	0·7	1·6	0·6	0·1	0·1	4·3	3·5	2·2	0·8	0	
Germany	5	100	13	1·6	0·6	1·6	0·6	0·2	0·1	1·9	0·8	1·4	0·6	0	
Italy	3	105	0	1·8	0·5	1·7	0·5	0		1·7	1·1	1·1	0·6	0	
Spain	1	20		0·9		0·9		0·1		2·7		1·9		0	
Sweden	2	105		1·4		1·2		0·2		2·1		1		0	
Average		105	33	1·5	0·6	1·3	0·5	0·1	0·1	2·6	1·7	1·5	0·7	0	
Rice drink															
Netherlands	5	222	13	1	0	0·1	0·1	0·2	0·1	10·2	0·7	5·3	1·4	0	
Belgium	6	197	13	0·8	0·3	0·1	0·0	0·1	0·1	9·6	0·9	5·3	1·1	0	
Germany	4	218	21	1	0·2	0·1	0·1	0·3	0·2	10	1·9	6	2	0	
Spain	8	218	21	1	0·2	0·1	0·1	0·2	0·2	9·3	3·7	5·6	1·3	0	
Italy	8	238	29	0·9	0·4	0·2	0·1	0·2	0·2	11·9	1·8	7·4	1·8	0	
Sweden	1	251		1		0·5		0·5		11		6		0	
Average		218	25	0·9	0·3	0·1	0·1	0·2	0·2	10·3	2·3	5·7	1·9	0	
Semi-skimmed. bovine milk															
Netherlands	13	197	4	1·5	0·1	1	0·1	3·5	0·3	4·8	0·2	0		4·7	0·4
Belgium	8	197	4	1·5	0·1)	1	0·04	3·4	0·2	4·9	0·1	0		4·8	0·1
Germany	19	197	4	1·5	0	1	0·2	3·4	0·1	4·9	0·1	0		4·9	0·1
Italy	30	197	8	1·7	0·0	1	0·1	3·3	0·3	4·9	0·1	0		4·9	0·1
Spain	29	192	4	1·6	0·0	1·1	0·1	3·2	0·2	4·7	0·1	0		4·7	0·1
Sweden	7	184	17	1·5	0	1	0·1	3·5	0·1	4·3	1	0		4·3	1
Average		197	8	1·6	0	1·0	0·1	3·3	0·3	4·8	0·3	0		4·7	0·3

Data are presented as means (sd).

Macronutrient compositions of the regular and organic varieties of all products were comparable (no further data shown). The unsweetened plant-based drinks contained no or very little sugar (average from 0·1 g/100 g for almond drink to 0·6 g/100 g for oat drink) and all unsweetened products had a lower caloric value (average ranged from 6 % less for rice to 40 % less for almond) than their respective regular types.

The average protein content of regular semi-skimmed bovine milk, across 6 countries, was 3·3 g/100 g (Table 2). Protein content of regular soy drink was on average 3·2 g/100 g. For the other regular plant-based drinks, the protein content was much lower and varied from 0·1 (coconut drink) to 0·8 (oat drink) g/100 g. Fat content in all drinks was comparable, around 2 g/100 g. SFA were highest in coconut drink (1·3 g/100 g) and bovine semi-skimmed milk (1·0 g/100 g). Regarding the carbohydrate- and sugar content, semi-skimmed bovine milk contained on average 4·7 g lactose/100 g. The regular varieties of plant-based drinks contained on average 2·1 (soy drink) to 5·7 (rice drink) g free sugar/100 g.

Bovine milk naturally contains micronutrients such as Ca, vitamin B_2_, vitamin B_12_ and I in significant quantities^(4)^. For the regular and unsweetened plant-based drinks, roughly half of the brands were fortified with Ca (Table 3). Among the brands which listed type of Ca, ∼60 % was fortified with calcium phosphate, and ∼40 % with calcium carbonate. Around 40 % of the brands was additionally fortified with vitamin B_12_ and vitamin D (Table 3). Vitamin B_2_ was added to approximately 30 % of the plant-based products. Iodine was only added to 1 product. The level of fortification was the same for nearly all plant-based drinks and was: 120 mg/100 g for Ca, 0·2 mg/100 g for vitamin B_2_, 0·4 µg/100 g for vitamin B_12_, 0·8 µg/100 g for vitamin D and 22 µg/100 g for I, which is similar to naturally occurring levels of Ca, vitamin B_2_, vitamin B_12_ and I in bovine milk. Bovine milk was not additionally fortified.

**Table 3:** Percentage of brands fortified with vitamin B_2_, vitamin B_12_, vitamin D, I and Ca in regular and organic drinks (as displayed on the nutritional label)

	Regular plant-based drinks											Organic plant-based drinks										
	Brands	Vitamin B_2_		Vitamin B_12_		Vitamin D		I		Ca		Brands	Vitamin B_2_		Vitamin B_12_		Vitamin D		I		Ca	
	*n*	% Fortified	sd	% Fortified	sd	% Fortified	sd	% Fortified	sd	% Fortified	sd	*n*	% Fortified	sd	% Fortified	sd	% Fortified	sd	% Fortified	sd	% Fortified	sd
Soy drink																						
Netherlands	4	100	0	100	0	75	50	0	0	100	0	2	0		0		0		0		50	71
Belgium	5	60	55	100	0	80	45	0	0	100	0	2	0		0		0		0		50	71
Germany	4	25	50	25	50	25	50	0	0	25	50	5	0		0		0		0		40	55
Italy	11	18	41	45	52	36	51	0	0	73	47	2	0		0		0		0		50	71
Spain	6	0	0	0	0	0	0	0	0	83	80	0										
Sweden	6	50	55	50	55	17	41	0	0	67	52	4	25	50	25	50	25	50	0	0	50	58
Average		36	49	50	51	36	49	0	0	75	44		7	26	7	26	7	26	0	0	47	52
Oat drink																						
Netherlands	5	33	52	33	52	33	52	0	0	33	52	4	0		0		0		0		0	
Belgium	7	29	49	71	49	57	58	0	0	71	49	5	0		0		0		0		0	
Germany	7	14	38	14	38	14	38	0	0	14	38	12	0		0		0		0		0	
Italy	8	38	52	50	54	50	54	0	0	50	54	6	0		0		0		0		0	
Spain	7	0	0	14	38	27	49	0	0	57	54	13	0		0		0		0		0	
Sweden	9	78	44	67	5	56	53	11	33	89	33	4	0		0		0		0		0	
Average		34	48	43	50	41	50	2	15	55	51		0		0		0		0		0	
Almond drink																						
Netherlands	6	67	52	67	52	67	52	0	0.	83	41	3	0		0		0		0		0	
Belgium	7	43	54	100	0	43	54	0	0	100	0	4	0		0		0		0		0	
Germany	7	14	38	14	38	14	38	0	0	29	49	8	0		0		0		0		0	
Italy	9	22	44	44	53	44	53	0	0	33	50	4	0		0		0		0		0	
Spain	10	0	0	50	53	60	52	0	0	70	48	4	0		0		0		0		0	
Sweden	3	0	0	0	0	0	0	0	0	0	0	3	0		0		0		0		0	
Average		24	43	50	51	43	50	0	0	57	50		0		0		0		0		0	
Coconut drink																						
Netherlands	5	20	45	100	0	60	55	0	0	100	0	3	0		0		0		0		0	
Belgium	3	0	0	33	58	33	0	0	0	33	58	2	0		0		0		0		0	
Germany	5	0	0	40	55	40	55	0	0	60	55	9	0		0		0		0		0	
Italy	3	0	0	0	0	0	0	0	0	0	0	1	0		0		0		0		0	
Spain	1	0		100		100		0		100		0										
Sweden	2	0		0		0		0		0		1	0		0		0		0		0	
Average		5	23	47	51	37	50	0	0	53	51		0		0		0		0		0	
Rice drink																						
Netherlands	5	0	0	40	55	40	55	0	0	40	55	1	0		0		0		0		0	
Belgium	6	0	0	83	41	67	51	0	0	83	41	6	0		0		0		0		0	
Germany	4	0	0	25	50	25	50	0	0	25	50	6	0		0		0		0		0	
Italy	8	13	35	25	46	2546)		0	0	50	53	8	0		0		0		0		0	
Spain	8	0	0	13	35	25	46	0	0	38	52	6	0		0		0		0		0	
Sweden	1	0		0		0		0		0		1	0		0		0		0		0	
Average		3	17	34	48	34	48	0	0	47	51		0		0		0		0		0	

Data are presented as percentage of fortified (sd).

Fortification practices varied per country and were observed to be relatively common in the Netherlands and Belgium, but were much less common in Germany and Spain (Table 3). In contrast to the regular plant-based drinks, the organic plant-based drinks were not fortified in any of the countries, except for a few organic soy drinks.

### Compositional analyses

In a representative selection of products, protein and amino-acid content was measured (Table 4). For the selected products, the average measured protein contents were similar to the values on the label. Measured protein content of fresh varieties was similar to the UHT varieties, except for oat drink and bovine semi-skimmed milk, in which protein content was ∼0·2 g/100 g lower in the fresh variety (data not shown).

**Table 4:** Protein and amino acid content, price and carbon footprint in a glass of 200 ml semi-skimmed bovine milk or plant-based drink (ultra-heat treatment)

	Semi-skimmed bovine milk		Soy drink		Oat drink		Almond drink		Coconut drink		Rice drink		*P*-value
	Mean	sd	Mean	sd	Mean	sd	Mean	sd	Mean	sd	Mean	sd	
Label values													
Energy (kJ)	404		324		386		186		168		418		
Protein (g)^1^	7·4		6		2		0·9		0·2		0·2		
Measured values:													
Protein	7·3	0·10	5·4**	0·05	2·4**	0·12	0·9**	0·02	0·2**	0·00	0·2**	0·01	<0·001
Total essential amino acids (g)	3·2	0·08	2·1**	0·01	0·8**	0·05	0·3**	0·01	0·1**	0·00	0·05**	0·00	<0·001
His (g)	0·170	0·004	0·129**	0·000	0·055**	0·003	0·012**	0·000	0·002*,**		0·009*,**		<0·001
Ile (g)	0·364	0·009	0·272**	0·001	0·101**	0·006	0·034**	0·001	0·007**	0·000	0·006**	0·000	<0·001
Leu (g)	0·670	0·016	0·417**	0·001	0·188**	0·011	0·060**	0·002	0·011**	0·001	0·010**	0·000	<0·001
Lys (g)	0·574	0·014	0·344**	0·001	0·089**	0·005	0·020**	0·001	0·007**	0·000	0·003**	0·000	<0·001
Met + Cys (g)	0·257	0·009	0·149**	0·002	0·133*,**		0·018**	0·002	0·006**	0·001	0·013**	0·000	<0·001
Phe + Tyr(g)	0·689	0·016	0·471**	0·002	0·232**	0·014	0·067**	0·002	0·010**	0·001	0·012**	0·000	<0·001
Thr (g)	0·394	0·009	0·202**	0·001	0·033**	0·002	0·004**	0·001	0·004**	0·000	0·001**	0·000	<0·001
Try (g)	0·096	0·002	0·076**	0·000	0·034**	0·002	0·020*,**		0·002**	0·000	0·003*,**		<0·001
Val (g)	0·440	0·011	0·272**	0·001	0·131**	0·008	0·037**	0·001	0·009**	0·000	0·007**	0·000	<0·001
Total non-essential amino acids	4·123	0·099	3·337**	0·010	1·534**	0·095	0·604**	0·016	0·515**	0·693	0·107**	0·007	<0·001
Ala	0·230	0·005	0·218	0·001	0·105**	0·006	0·039**	0·001	0·009**	0·001	0·009**	0·000	<0·001
Arg	0·230	0·005	0·399**	0·001	0·151**	0·009	0·084**	0·002	0·023**	0·001	0·018**	0·000	<0·001
Asp	0·536	0·013	0·635	0·002	0·198**	0·012	0·094**	0·002	0·016**	0·001	0·015**	0·000	<0·001
Glu	1·513	0·036	1·070**	0·003	0·555**	0·034	0·235**	0·006	0·037**	0·002	0·027**	0·001	<0·001
Gly	0·132	0·003	0·218**	0·001	0·117	0·007	0·052**	0·001	0·009**	0·001	0·009**	0·000	<0·001
Pro	0·689	0·016	0·272**	0·001	0·133**	0·008	0·035**	0·001	0·005**	0·000	0·007**	0·000	<0·001
Ser	0·383	0·009	0·272**	0·001	0·107**	0·007	0·034**	0·001	0·008**	0·000	0·007**	0·000	<0·001
% of WHO requirements													
His (%)	24·3	0·6	18·4**	0·1	7·9**	0·5	1·8**	0·0	0·4**	0·0	1·2**	0·1	<0·001
Ile (%)	26·0	0·6	19·4**	0·1	7·2**	0·4	2·4**	0·1	0·5**	0·0	0·4**	0·0	<0·001
Leu (%)	24·5	0·6	15·3**	0·1	6·9**	0·4	2·2**	0·1	0·4**	0·0	0·4**	0·0	<0·001
Lys (%)	27·4	0·7	16·4**	0·1	4·2**	0·3	1·0**	0·0	0·3**	0·0	0·2**	0·0	<0·001
Met + Cys (%)	24·4	0·9	14·2**	0·2	12·6**	0·8	1·7**	0·1	0·5**	0·0	1·2**	0·1	<0·001
Phe + Tyr (%)	39·4	0·9	26·9**	0·1	13·2**	0·8	3·8**	0·1	0·6**	0·0	0·7**	0·0	<0·001
Thr (%)	37·6	0·9	19·2**	0·1	3·1**	0·2	0·4**	0·0	0·4**	0·0	0·1**	0·0	<0·001
Trp (%)	34·2	0·8	27·2**	0·1	12·0**	0·7	7·2**	0·2	0·6**	0·2	1·2**	0·1	<0·001
Val (%)	24·2	0·6	14·9**	0·1	7·2**	0·4	2·0**	0·1	0·5**	0·0	0·4**	0·0	<0·001
Minimum	24·2	0·6	14·2**	0·2	3·1**	0·2	0·4**	0·0	0·3**	0·0	0·1**	0·0	<0·001
Price (€)	0·26		0·30		0·40		0·52		0·46		0·33		
Greenhouse gas emission (gram CO_2_-equivalents)	312		88		60		93		101		149		

Data are presented as means (sd).

* The detection limit has been used as amino acid value.

** The protein or essential amino acid content is significantly different from bovine milk (*P* < 0·001)

Protein content was highest in bovine milk. For essential amino acid content, the difference with plant-based drinks was even larger, as in these analyses not only protein quantity, but also protein quality was accounted for. One glass of bovine milk (200 ml) contained at least 24 % of the WHO requirements for each of the essential amino acids. For the plant-based drinks the contribution to the required level was significantly lower: 14·2 % for soy drink, 3·1 % for oat drink, 0·4 % for almond drink, 0·3 % for coconut drink and only 0·1 % for rice drink. Of all drinks, bovine milk was the cheapest product. Carbon footprint was lowest for plant-based drinks. Among the plant-based drinks, oat drink had the lowest carbon footprint.

Table 5 describes the interrelationship between essential amino acids, kJ, price and greenhouse emission. Table 5 showed that in order to consume at least 24 % of WHO requirements for each essential amino acid, representative for 1 glass of semi-skimmed milk, consumption of 1·7 (soy drink) to 246·3 (rice drink) glasses of plant-based drinks are required. Semi-skimmed bovine milk provided the lowest amount of energy and required the least amount of money to provide at least 24 % of WHO requirements for each of the essential amino acids. Soy drink scored best on carbon footprint and required 160 g CO_2_-equivalents to provide at least 24 % of WHO requirements for each essential amino acid. For semi-skimmed bovine milk, this was 312 g CO_2_-equivalents and for oat drink 474 g CO_2_-equivalents. For the other plant-based drinks, greenhouse gas emission was above 5000 g CO_2_-equivalents when meeting essential amino acids requirements.

**Table 5:** kJoules, price and greenhouse gas emission to provide at least 24 % of the WHO requirements* for each essential amino acid

	Semi-skimmed. bovine milk	Soy drink	Oat drink	Almond drink	Coconut drink	Rice drink
Glasses (of 200 ml)	1	1·7	7·9	58·2	70·6	246·3
Energy (kJ)	404	554·8	3040·9	10714·4	11815·6	103051·9
Price (€)	0·3	0·5	3·1	30·1	32·3	81·3
Greenhouse gas emission (gram CO_2_-equivalents)	312	149	476	5436	7159	36 797

* The amino acid with the lowest contribution to the essential amino acids requirements is rate limiting for protein synthesis. One glass of semi-skimmed bovine milk contains at least 24 % of the WHO requirements for each of the essential amino acids.

## Discussion

Semi-skimmed bovine milk naturally contains high quality protein and many different micronutrients, such as Ca, vitamin B_2_, vitamin B_12_ and I. The content of protein and essential amino acids in bovine milk were higher than in any of the plant-based drinks. As a relatively large percentage of the plant-based drinks, especially the organic varieties, were not fortified, also micronutrients are an important discriminating factor between bovine milk and plant-based drinks. Per litre, the carbon footprint of bovine milk was higher than the carbon footprint of plant-based drinks. However, given the market positioning, it is important to consider the nutrition dimension as well. When accounting for protein quality, the carbon footprint of bovine milk was, except for soy drink, lower than the carbon footprint of plant-based drinks.

In the current study, nutrition labels of semi-skimmed bovine milk and plant-based drinks of 6 Europeans countries were systematically analysed on macro- and micronutrient content. For soy drink, the nutritional values on the labels were in line with values reported in national food composition databases. For the other plant-based drinks, the food composition databases did not report an average nutrient content value. According to the labels, macronutrient composition differed between bovine milk and plant-based drinks for protein, SFA and free sugar (Table 2). Semi-skimmed bovine milk contained ∼3·3 g protein/100 g and soy drink ∼3·2 g protein/100 g. For all other plant-based drinks, the protein content was on average below 1 g/100 g. In line with other studies^(3,7,10–12)^, this study showed that, based on declared total protein content, soy drink is a reasonable substitute for bovine milk, but the other plant-based drinks are not. Protein quality cannot be declared on the label. However available literature^(7,43)^, as well as the measurements performed in this study (Table 4), show that bovine milk protein has a higher protein quality than plant-based protein.

The above-mentioned differences in protein quantity and quality are especially relevant for population groups with an increased protein need and/or a low protein intake such as elderly, children and people who eat no, or a limited amount, of animal-based products. For elderly, who generally have an increased protein need and low protein intake, it is important to consume products rich in high quality protein in order to prevent sarcopenia^(14)^. Because of growth, children have increased protein needs. Accordingly, Morency *et al*.^(15)^, showed that children, who consumed plant-based drinks were generally shorter. Finally protein quantity and quality are of concern in people who eat no, or a limited amount, of animal-based products, like meat, and hence have a lower protein and essential amino acid intake^(30)^.

Saturated fatty acid content also differed between bovine milk and plant-based drinks. SFA were highest in coconut drink (1·4 g/100 g), while for semi-skimmed bovine milk they were 1·0 g/100 g. For other plant-based drinks, saturated fatty acid content was at or below 0·4 g/100 g. SFA have been shown to increase LDL cholesterol levels. Although this latter association is established, a systematic review showed no association of dairy foods with increased cholesterol levels or with CVD. On the contrary, some studies have shown a protective association between dairy products and cardiovascular outcomes, such as stroke and hypertension^(31)^.

The final macronutrient with a significant different content in bovine milk and plant-based drinks is free sugar. Although on the label no distinction is made between free and non-free sugars, WHO makes a clear difference between those sugar types. According to WHO, free sugars are defined as added sugar, as well sugars formed during the production process^(19)^. Free sugars, particularly in the form of sugar-sweetened beverages, are associated with a higher body weight^(19)^. Furthermore, free sugar is associated with dental caries^(19)^. On the other hand, regarding sugars intrinsically present in milk, such as lactose, WHO states that there is no reported evidence of adverse effects^(19)^. Plain semi-skimmed, bovine milk does not contain any free sugar. All sugars in the plant-based drinks are either added or formed during the production process, and thus considered by the WHO definition as free sugars. The regular varieties of plant-based drinks contained on average 2·1 (soy drink) to 5·9 (rice drink) g free sugar/100 g. This is lower than average sugar level in for example juice or soda, which all contain around 10 g free sugar/100 g^(6)^. However, as the intake of free sugar is generally too high in Western countries^(32)^, drinks with low or no free sugar could help to limit sugar intake.

Regarding micronutrients, bovine milk differs from plant-based drinks (Table 3). Milk naturally contains many micronutrients such as Ca, I, vitamin B_2_ and vitamin B_12_, all in a highly bioavailable delivery matrix^(33)^. Data in the current study showed that for regular plant-based drinks, roughly 50 % of the products is fortified with Ca and roughly 40 % is additionally fortified with 1 or more other micronutrients. For the organic plant-based drinks, hardly any is fortified. The latter can be explained by EC regulation, which only allows fortification of organic foods with vitamins and minerals, as far as their use is legally required^(34)^. The fact that a large percentage of the plant-based drinks portfolio is unfortified and that intake of ‘dairy nutrients’ by other food groups is in several cases insufficient to meet nutrient recommendations, means that replacement of bovine milk by these unfortified drinks carries the risk of suboptimal nutrient intake and deficiencies for Ca and B vitamins. The impact of such deficiency on bone health and other health outcomes, would be particularly apparent in sensitive groups such as children, elderly and vegans.

Despite being established that dairy makes a major contribution to total I intake^(5)^, it is remarkable that hardly any plant-based drink has been fortified with I. This may partly explain the increased frequency of suboptimal I intake and I deficiency in people who eat no or limited animal-source foods^(35)^.

The nutritional information above, which has been based on, or derived from, nutritional labels, gives a good insight on the nutritional value of milk and plant-based drinks. However, to determine the position of milk and plant-based drinks in a sustainable diet, one would need information on a sustainability parameter like carbon footprint. Furthermore, it would be best, rather than considering only the protein quantity, to use data on protein quality as well. To the best of our knowledge, this is first study to have measured essential amino acids and to have determined carbon footprint in semi-skimmed bovine milk and plant-based drinks.

Among the drinks selected, the semi-skimmed bovine milk had the highest protein content. For total essential amino acid content, the difference between bovine milk and plant-based drinks was larger (Table 4). The difference became even larger, if in addition the WHO amino acid requirements were accounted for. This means that bovine milk contains more protein, more essential amino acids and has better ratio among the different essential amino acids. This result on protein quality of bovine milk is confirmed by studies on ingredients, which show that the DIAAS value of dairy protein ingredients, such as milk protein concentrate, is higher than that of plant protein ingredients, such as soy and rice isolates^(11)^.

Regarding climate impact, plant-based drinks had a lower carbon footprint than semi-skimmed bovine milk. However, the carbon footprint should be assessed in the context of the nutritional value, i.e., in the context of a sustainable diet. A sustainable, healthy diet will among others, provide sufficient micronutrients, as well as essential amino acids.

From a micronutrients perspective, it is important that plant-based drinks, which are positioned as dairy alternatives, should provide similar vitamins and minerals. As plant-based drinks naturally contain no or very limited ‘dairy micronutrients’, the unfortified alternatives are not eligible for a place in a sustainable, healthy diet.

The key nutritional differentiator between bovine milk and fortified plant-based drinks is milk’s high-quality protein. As in addition protein is also the main determinant of carbon footprint, it is important to consider the balance between protein quality and carbon footprint. One glass of semi-skimmed bovine milk has a carbon footprint of 312 g CO_2_-equivalents. In order to provide a similar protein quality one would need to drink so many glasses of almond, rice or coconut drink, that their accumulative carbon footprint would be above 5000 g CO_2_-equivalents (Table 5). Therefore, for those drinks, the nutrition *v*. climate change balance is so far off, that a significant place in a sustainable diet cannot be justified.

For soy and oat drink, it was shown that 1·7 glasses of soy drink (142 g CO_2_-equivalents) or 7·9 glasses of oat drink (476 g CO_2_-equivalents) are required to provide a similar protein quality as milk.

Fortified soy drink scores best on the nutrition to climate change balance, and semi-skimmed milk scores second best. The latter conclusion is in line with the study of Tessari *et al.* ^(36)^, which compared milk with soy beans.

However, as stated earlier, a sustainable diet also accounts for economic and social factors^(17)^. Compared with soy drink, semi-skimmed bovine milk has a higher consumer acceptance, due to e.g. its lower price, better fit with current consumption habits and better taste^(37,38)^. All in all it can be concluded that for both bovine milk and fortified (but not unfortified) soy drink, there is a place in a sustainable diet. This conclusion is in line with several other studies, including the renowned EAT-Lancet study, which shows that bovine milk has a place in a sustainable diet^(2,39,40)^. Furthermore the conclusion is line with the American Dietary Guidelines 2020–2025, in which it is stated that among the plant-based drinks, fortified soy drink, is the only acceptable dairy alternative^(41)^.

Regarding the nutrition *v*. sustainability balance, fortified oat drink comes on the third place, after fortified soy drink and bovine milk. It scores much better than almond, coconut and rice drink, but the gap with bovine milk and fortified soy drink is still significant. In order to deserve a significant place in a sustainable diet, the nutrition *v*. sustainability balance of fortified oat drink would need an upgrade. This could, for example, be done by adding a nutritionally complementary plant protein source such as pea, to achieve a better balance of essential amino acids. However to realise this for liquid drinks, several technical issues, such as issues with colour and taste, should be resolved first^(42)^.

Finally, it is important to acknowledge that although the scope of the current study is limited to a direct product comparison, also the broader dietary context should be considered. A study by Sonesson *et al*.^(43)^ found that the importance of different protein foods for essential amino acid provision, as assessed by the Protein Quality Index, did not differ significantly between a habitual, low-meat or vegetarian Swedish diet, although this might change in a more extreme dietary scenario. The current sustainable diet recommendations would be comparable with the vegetarian or low meat scenario^(3,39,40)^. In a more extreme scenario, in which additionally dairy protein is reduced and not adequately replaced, recommendations for total nitrogen and essential amino acids, like lysin, may not be met^(44,45)^. The ability of plant-based drinks to adequately replace dairy protein, will partly depend on co-ingestion of other foods with a complementary amino acid profile. The latter will be most relevant for plant-based drinks with a relatively low protein quality, but with reasonable protein level, e.g. oat drink.

This study contains several strengths and limitations. Nutritional labels of 6 European countries were analysed in a systematic, unbiased way. However, nutritional information on protein quality is not reported on the label. To get a more detailed insight, the amino acid composition was measured in a selection of Dutch products. Protein content was measured and amino acid per gram protein was determined. Another strength is the calculation of carbon footprint. Rather than obtaining carbon footprint data from different references, carbon footprints were calculated with the latest LCA methodology for all products, in which essential amino acids had been measured. For plant-based drinks, LCA’s were based on publicly available data rather than production data, and hence some assumptions had to be made. However, in case the supplier had provided a carbon footprint of their end product, the numbers matched the number calculated in this paper.

Unlike for carbon footprint, data needed to make a calculation for other environmental parameters were not all publicly available. Based on other literature^(36,43)^, one would expect that the difference between bovine milk and plant-based drinks would be somewhat smaller for land use than for carbon footprint. Furthermore, essential amino acids and carbon footprint data were obtained in a selection of Dutch food products. As the nutritional label declaration was broadly similar across Europe, conclusions are expected to be more or the less the same across Europe. Small deviations could occur due to the relatively high protein content of Dutch milk (Table 2), which could have increased both nutritional value and carbon footprint. Differences in origin of plant-based ingredients, amount of heat and electricity in processing, utility technology mix used, as well differences in transport distance to and from the factory, could have caused some variation in the carbon footprint values. Furthermore, the carbon footprint for the average oat drink is expected to be a bit higher, as the selected oat drink brand had a relatively low carbon footprint. Finally, from a public health perspective, a comparison on health benefits, i.e. disability adjusted life year (DALY’s), would be desirable. Although for dairy estimates on the contribution to DALY’s exist^(46)^, the association between plant-based drinks and health has hardly been investigated^(47)^. Therefore, such a comparison is not feasible.

In conclusion, manufacturers produce plant-based drinks with a similar appearance as bovine milk and position them as milk alternatives. This study shows that the nutritional composition of plant-based drinks differs significantly from bovine milk. It is important to account for those nutritional differences in dietary advises. The latter is especially important for vulnerable groups, such as children and elderly. Considering the increasing importance of climate change, in addition to nutritional value, also climate impact should be considered. Overall, it can be concluded, that the nutrition–climate change balance presented in this study, is in line with previous literature, which shows that both semi-skimmed bovine milk and fortified (but not unfortified) soy drink deserve a place in a sustainable diet.
